# Electrical performance of a nanocomposite diode based on palladium nanoparticles- and polyethyleneimine functionalized nitrogen-doped graphene quantum dots

**DOI:** 10.1038/s41598-025-25597-x

**Published:** 2025-11-07

**Authors:** Elanur Dikicioğlu, Halil İbrahim Efkere, Mustafa Yıldız, Elif Orhan

**Affiliations:** 1 Electroneurophysiology Program, Yüksek İhtisas University, 06800 Ankara, Turkey; 2 Photonics Research Center, Gazi University, 06500 Ankara, Turkey; 3 Department of Chemistry, Çanakkale Onsekiz Mart University, 17100 Çanakkale, Turkey; 4 Department of Physics, Gazi University, 06500 Ankara, Turkey

**Keywords:** Graphene quantum dots, Nitrogen doping, Palladium nanoparticles, Nanocomposite diode, Surface functionalization, Chemistry, Materials science, Nanoscience and technology, Physics

## Abstract

A nanocomposite material comprising palladium nanoparticles (PdNPs) doped with polyethyleneimine (PEI)-functionalized nitrogen-doped graphene quantum dots (PEI N-GQDs) was synthesized and employed as an interlayer in an Ag/PdNPs/PEI N-GQDs/p-Si heterojunction configuration. The PdNPs/PEI N-GQDs nanocomposite was prepared through an aqueous reaction between PEI-functionalized N-GQDs and Pd(NO_2_)_2_·H_2_O and characterized using FTIR, UV–Vis, TEM, XPS, and PL spectroscopy. The PL spectrum exhibited a broad emission centered at 580 nm (2.14 eV) with a near-infrared tail, indicating defect-assisted recombination and confirming the semiconducting nature of the nanocomposite. The Ag/PdNPs/PEI N-GQDs/p-Si diode displayed a rectification ratio of approximately 1.64 × 10^2^ at ± 4 V, with barrier heights of 0.87, 0.76, and 0.68 eV obtained from thermionic-emission (TE), Cheung, and Norde methods, respectively. Energy band alignment analysis revealed that the rectifying behavior originates from the Ag/nanocomposite interface, forming a Schottky-type barrier, while a type-II staggered junction is established at the nanocomposite/p-Si interface, facilitating hole transport. These findings demonstrate the potential of PdNPs/PEI N-GQDs as an effective interlayer material for carbon-based nanoelectronic and optoelectronic devices.

The development of semiconductor-based electronic devices remains a cornerstone of technological advancement. In recent years, carbon-based nanomaterials have emerged as attractive alternatives or complementary components to conventional metals and inorganic semiconductors. Among these, carbon allotropes and their derivatives—such as carbon nanotubes (CNTs), graphene sheets, fullerenes, and graphene quantum dots (GQDs)—have garnered significant attention in photodetection and electronics due to their tunable bandgap, high light absorption, and excellent carrier mobility^1–3^.

GQDs are quasi-zero-dimensional (0D) carbon nanostructures characterized by quantum confinement and edge effects, providing exceptional chemical stability, broad bandgap tunability, and strong photostability^4^. Their large surface area also enables facile functionalization and integration with other materials, allowing precise control over optical and electronic properties depending on the application^5–7^. To regulate these properties, various doping strategies have been developed^8–10^. Heteroatom doping, particularly N doping, modifies the band structure and enhances the conductivity of GQDs by introducing n-type behavior and increasing electron density^11–13^.

Surface functionalization plays a similarly important role in enhancing performance. Polyethyleneimine (PEI), a polymer rich in amine groups, improves water solubility, supports uniform thin-film formation, and creates a positive surface dipole layer that alters energy alignment at interfaces^14–16^. The combination of nitrogen doping and PEI functionalization (referred to as PEI@N-GQDs) enables high-quality film fabrication and interfacial engineering on semiconductor surfaces^17^. PEI@N-GQDs thin films deposited on n/p-type Si wafer have been successfully employed in Schottky diode structures, and their electrical characteristics have been systematically investigated both theoretically^18,19^ and experimentally^20–25^.

Beyond heteroatom doping, rare-earth elements have also been used to further enhance the optical and electronic properties of GQDs. Lanthanide-based dopants such as Lanthanum (La), Gadolinium (Gd), and Europium (Eu) are known to improve physical performance in carbon-based nanostructures, leading to the formation of new functional hybrids for optoelectronic applications^20–22,26^. For instance, La(OH)_3_ nanoparticles incorporated into PEI@N-GQDs have demonstrated enhanced photodetection and modulated Schottky barrier characteristics^20^. Gd-doped PEI@N-GQD Schottky diodes, in comparison to undoped controls, exhibited drastic changes in rectification behavior—reducing the rectification ratio from ~ 2.8 × 10^4^ to ~ 14—suggesting new charge transport paths or interfacial states^22^. Similarly, Europium (Eu) and Terbium (Tb) doped carbon dots have shown promising photoluminescence and magnetic properties for Magnetic Resonance Imaging (MRI) and bioimaging applications^22,23^. Despite these advances, the impact of rare-earth dopants on diode performance remains underexplored^17,20,21^.

In parallel, transition metal dopants have drawn growing interest due to their rich d-band structures and multiple oxidation states, enabling fine-tuning of material behavior. Noble metals such as Gold (Au), Platinum (Pt), and Palladium (Pd) demonstrate excellent electronic and catalytic properties when integrated into carbon-based systems^27,28^. For example, Au and silver (Ag) nanoparticles have improved optical responsivity in graphene-based platforms via localized surface plasmon resonance^28^, while Pt-doped PEI@N-GQD composites have enhanced reactivity and decreased energy gaps in sensor applications^29^. Palladium (Pd), with its high work function (~ 5.1 eV) and hydrogen affinity, has been used in graphene- and CNT-based gas sensors to improve conductivity and surface activity^30,31^. Pd-loaded PEI@N-GQDs has also been explored in biomedical contexts, revealing notable effects on cellular interaction and potential in cancer therapy^32^. However, the application of Pd doping in Schottky diodes incorporating GQDs remains largely unexplored.

A novel nanocomposite material, PdNPs/PEI N-GQDs, was synthesized and integrated into an Ag/p-Si heterojunction to address this research gap. The structural and electrical characteristics of the resulting Schottky diode were systematically investigated to assess its suitability for future carbon-based electronic applications (Fig. 1).

**Fig. 1:**
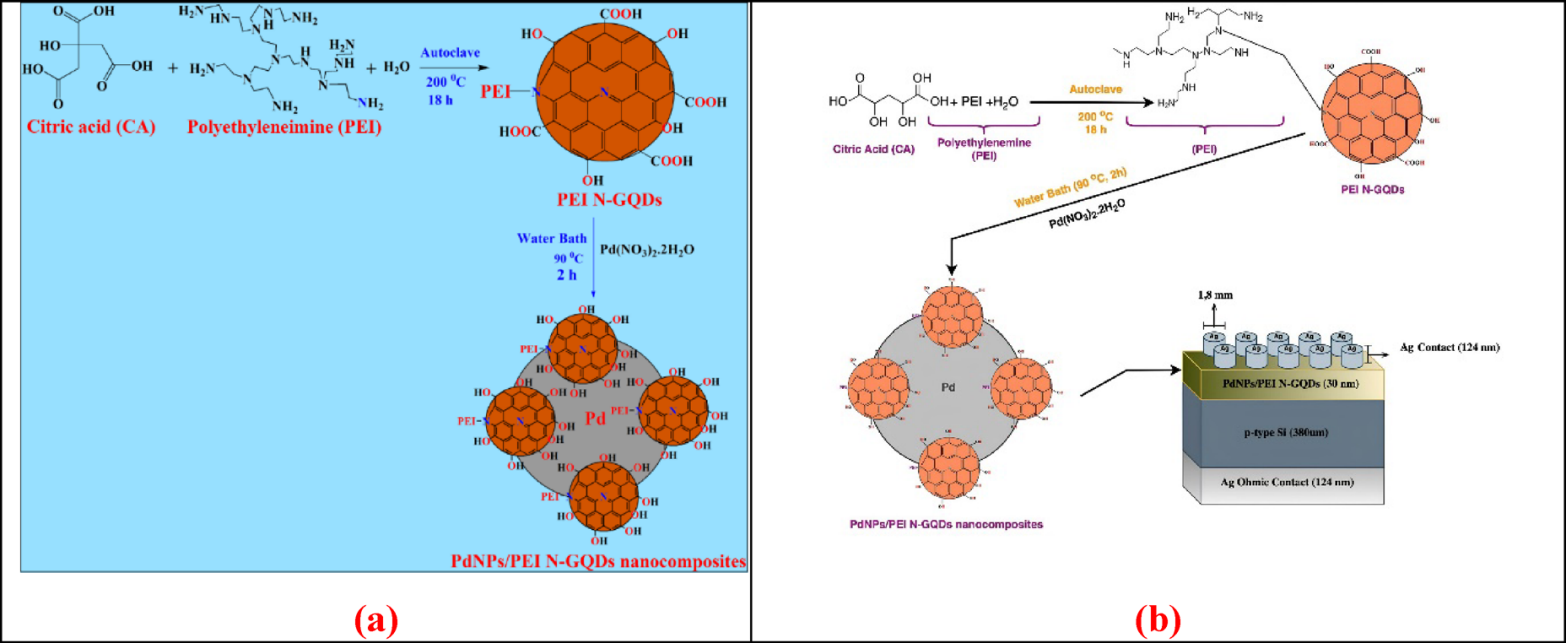
(**a**) Schematic illustration of the synthesis of PEI N-doped GQDs and the PdNPs/PEI N-GQDs nanocomposite; (**b**) schematic representation of the Ag/PdNPs/PEI N-GQDs/p-Si heterojunction structure.

## Results

### Characterization of PdNPs/PEI N-GQDs nanocomposites (FT-IR, UV-Vis, TEM and XPS)

**Fig. 2:**
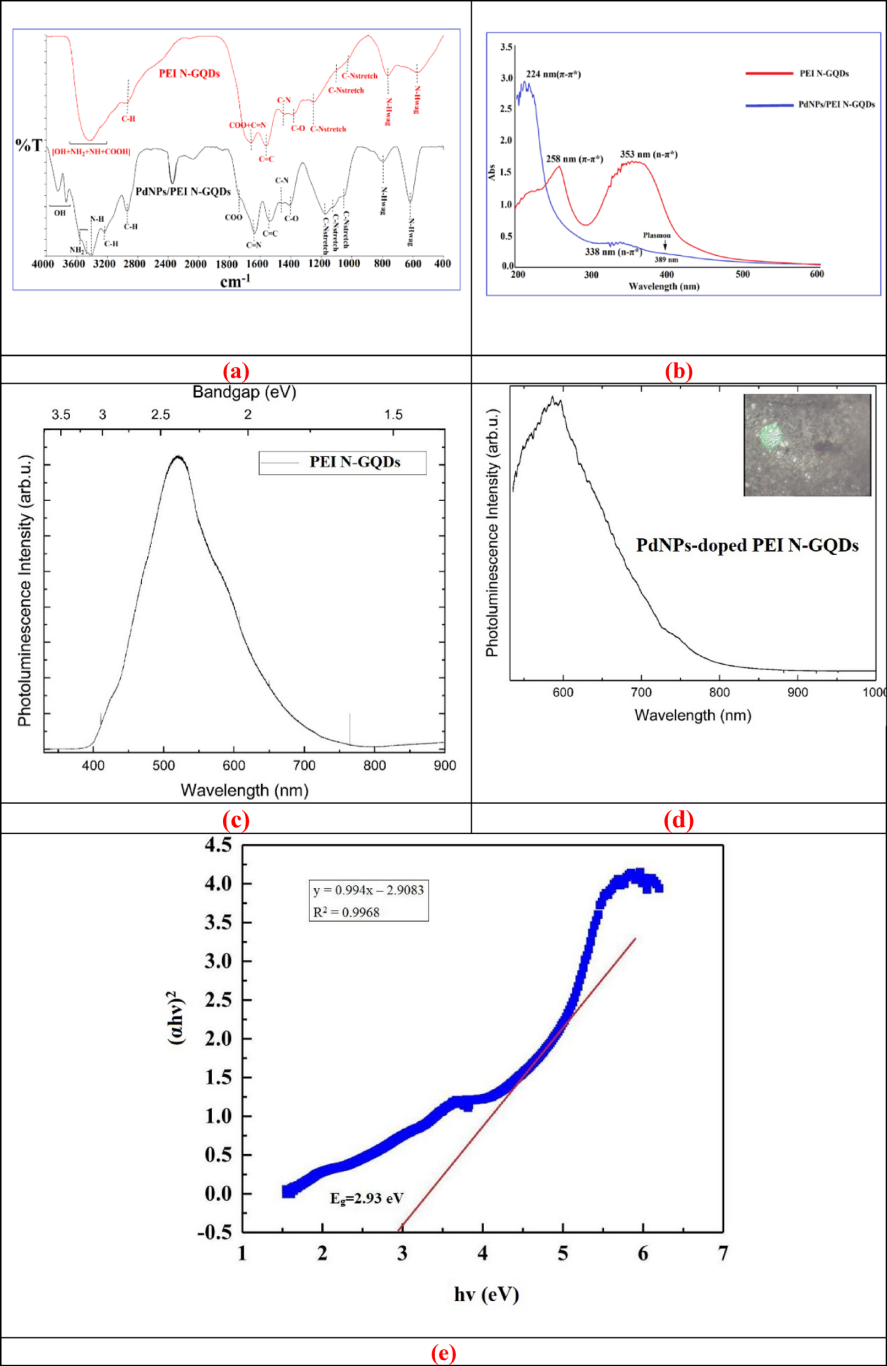
(**a**) FTIR spectra of PEI N-GQDs and PdNPs-doped PEI N-GQDs nanocomposites, showing transmittance (%T) as a function of wavenumber (cm^− 1^), (**b**) UV–Vis spectra of PEI N-GQDs and PdNPs-doped PEI N-GQDs nanocomposites as a function of wavelength (nm), and (**c**) PL spectrum of pristine PEI N-GQDs solution as a function of wavelength (nm) and bandgap energy (eV), and (**d**) PL spectrum of PdNPs-doped PEI N-GQDs nanocomposite. (The inset displays the optical microscope image of the sample under excitation). (**e**) Tauc plot of (αhν)^2^ versus photon energy (hν) for the PdNPs-doped PEI N-GQDs nanocomposite.

Fourier Transform-Infrared (FT-IR) spectra of the starting compound PEI N-GQDs and PdNPs/PEI N-GQDs nanocomposites are given in Fig. 2a, showing transmittance (%T) as a function of wavenumber (cm^−1^). When PdNPs/PEI N-GQDs nanocomposites were compared to the starting compound PEI N-GQDs, different distinctive vibrations were recorded, indicating the formation of Pd nanocomposites. The OH + NH_2_ + NH + COOH, C–H, O–C = O + C = N(pyridinic), C=C, C–N, and C–O functional group vibrations in PEI N-GQDs material were observed at 3433 cm^− 1^ strong-broad, 2931 cm^− 1^ strong, 1702–1654 cm^− 1^ strong-broad, 1557 cm^− 1^ strong, 1440 cm^− 1^ medium and 1302 cm^− 1^ medium, respectively^18,20,22,29,32–35^. For PEI N-GQDs, the C–N stretching and N–H wagging vibrations were observed at 1252, 1081, and 1020 cm^− 1^, and at 772 and 568 cm^− 1^, respectively. In the PdNPs/PEI N-GQDs nanocomposite, these vibrations appeared at 1169, 1112, and 1046 cm^− 1^ for C–N stretching, and at 785 and 616 cm^− 1^ for N–H wagging. Accordingly, in the PdNPs/PEI N-GQDs nanocomposite, the C–N stretching vibrations shifted to lower frequencies, while the N–H wagging vibrations shifted to higher frequencies. In the FT-IR spectrum of PEI N-GQDs, the vibrations of (OH + NH_2_ + NH + COOH) and (O–C = O + C = Npyridinic) groups overlapped^18,20,22,29,32–35^.

It was observed that the vibrations of all functional groups in PdNPs/PEI N-GQDs nanocomposites were shifted to higher frequencies than PEI N-GQDs. These results suggest that PEI N-GQDs are oxidized and that Pd(II) metals are reduced^22,29,32,33^.

Comparative Ultraviolet-Visible (UV–Vis) spectra as a function of wavelength (nm) were recorded for PEI N-GQDs and PdNPs/PEI N-GQDs nanocomposites in water (Fig. 2b. Two bands, which are assigned to π–π* transitions of C=C and n–π* transitions of C=N and C=O, were observed at 258 and 353 nm in the UV–Vis spectrum of PEI N-GQDs (Fig. 2b)^18,20,22,29,32–35^. In the PdNPs/PEI N-GQDs nanocomposites, the π–π* and n–π* transitions were found to occur at wavelengths of 224 nm and 338 nm, respectively. In the PdNPs/PEI N-GQDs nanocomposites, the π–π* and n–π* transitions were found to occur at wavelengths of 224 nm and 338 nm, respectively. They were also assigned to the shoulder plasmon resonance at 389 nm (Fig. 2b)^22,29,32,33^.

**Fig. 3:**
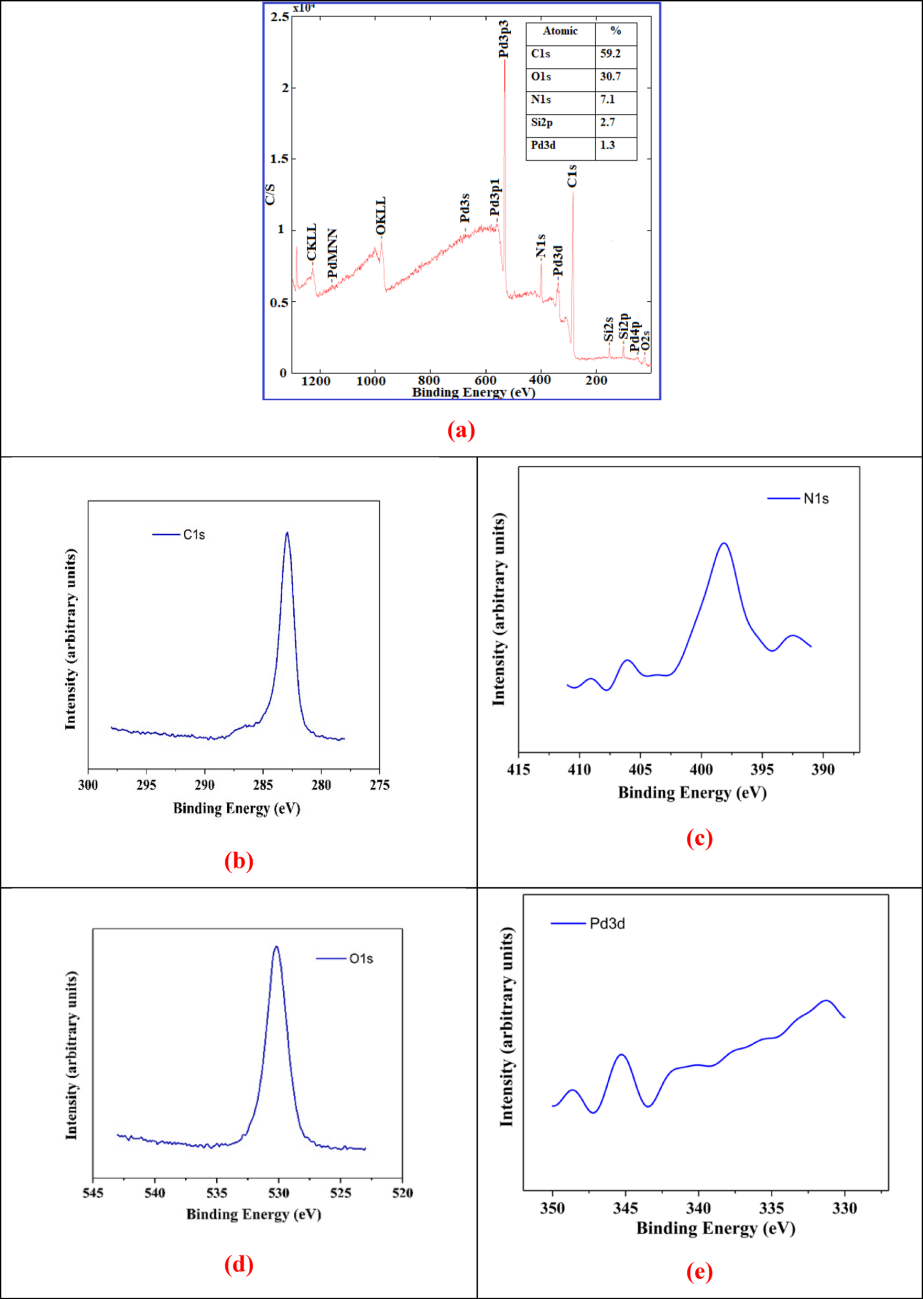
(**a**) Survey XPS spectrum of PdNPs/PEI N-GQDs; high-resolution XPS spectra of PdNPs/PEI N-GQDs for (**b**) C 1s, (**c**) N 1s, (**d**) O 1s, and (**e**) Pd 3d core levels.

Beyond the electrical characterization of the fabricated diode, the photoluminescence (PL) response of the PdNP-doped PEI N-GQDs was investigated to gain complementary insights into the influence of Pd incorporation on the nanocomposite’s optical behavior. Figure 2c presents the PL spectrum of pristine PEI N-GQDs, whereas Fig. 2d shows that of the PdNP-doped nanocomposite. The Pd-doped sample exhibits a broad emission band centered around 580–600 nm with a long tail extending toward the near-infrared (NIR) region (700–900 nm). Compared to pristine GQDs, the PdNP-doped GQDs display a pronounced red-shift and spectral broadening, suggesting the formation of defect-related states and enhanced charge-transfer interactions between Pd nanoparticles and GQD surface sites. The inset image highlights localized emissive regions on the sample surface, further supporting the presence of non-uniform defect distributions.

These observations indicate that Pd doping significantly modifies the electronic and optical structure of GQDs by promoting defect-assisted luminescence in addition to band-edge recombination. To further evaluate the optical band structure, the absorption behavior of the PdNPs-doped PEI N-GQDs nanocomposite was analyzed using the Tauc relation. Figure 2e shows the Tauc plot of (αhν)^2^ versus photon energy (hν), assuming a direct allowed transition. The extrapolation of the linear region to the energy axis yields an optical band gap (E_g_) of approximately 2.93 eV. The lower PL emission energy (~ 2.14 eV, 580 nm) compared to the optical band gap reflects radiative recombination through defect and surface states. Overall, the optical characterization confirms the semiconducting nature of the nanocomposite and evidences that Pd incorporation alters the band structure through defect formation and charge-transfer coupling.

The elemental composition and binding configuration of the PdNPs/PEI N-GQDs nanocomposite were analyzed using X-ray Photoelectron Spectroscopy (XPS) (Fig. 3)^18,20,22,29,32–35^. From the XPS spectra, the C, N, O and Pd binding energies of PdNPs/PEI N-GQDs nanocomposites were 284.37(C1s), 399.37(N1s), 531.37(O1s), and 674.37(Pd3s), 557.37(Pd3p_1/2_), 533.37(Pd3p_3/2_), 342.37(Pd3d_5/2_), 338.37(Pd3d_5/2_), 334,37(Pd3d_5/2_) eV, respectively. Furthermore, C, Pd, and O Auger peaks (CKLL, PdMNN, and NKLL) are observed at 1226.37, 1157.37, and 977.37 eV, respectively. Figure 3a,b–e) present the XPS spectra of C 1s, N 1s, O 1s, and Pd 3d, confirming the formation and bonding configurations of the PdNPs/PEI N-GQDs nanocomposite^22,29,32,33^.

**Fig. 4:**
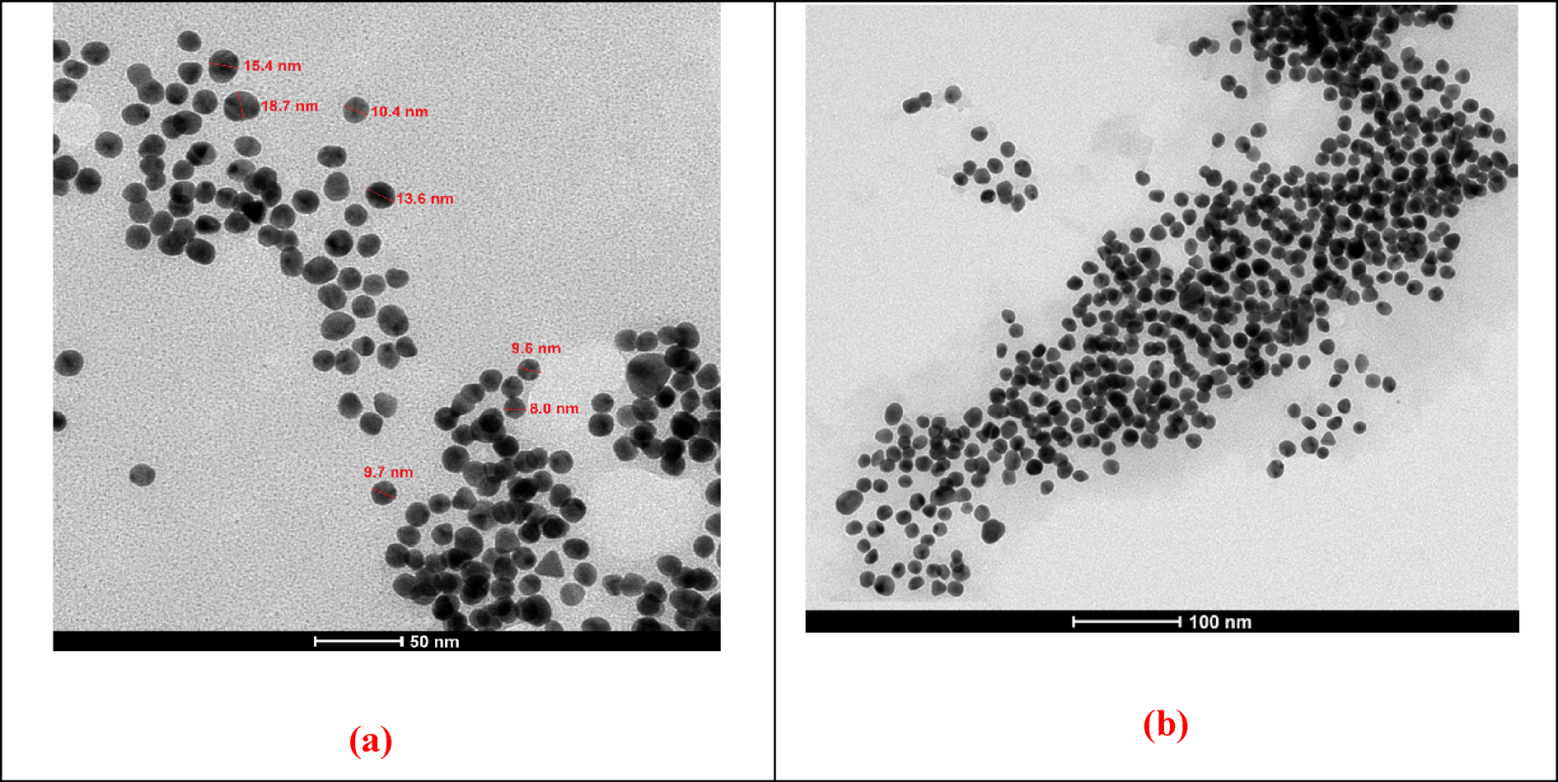
TEM images of PdNPs/PEI N-GQDs nanocomposites at different magnifications: (**a**) 50 nm and (**b**) 100 nm.

The structure of PdNPs/PEI N-GQDs nanocomposites was investigated by using Transmission Electron Microscopy (TEM) analysis, and the corresponding images are shown in Fig. 4a,b^22,30,33,34^. TEM images at 50 and 100 nm magnifications reveal that Pd forms spherical, porous structures with an average size of 8.0–18.7 nm, without noticeable agglomeration (Fig. 4a,b).

### Electrical performance of Ag/ PdNPs/PEI N-GQDs /p-Si heterojunction diode

To evaluate the charge transport characteristics of the Ag/PdNPs/PEI N-GQDs/p-Si diode, Thermionic Emission (TE) theory was applied under forward bias conditions. According to TE theory, the current passing through a Schottky diode and the forward bias voltage (*V > 3kT/q*) have the following relationship^36,37^

1 $$I = I_{0} \exp \left( {\frac{{qV}}{{nkT}}} \right)\left[ {1 - \exp \left( { - \frac{{qV}}{{kT}}} \right)} \right]$$

2 $$I_{0} = AA^{*} T^{2} \exp \left( { - \frac{{q\varphi _{b} }}{{kT}}} \right)$$

In Eqs. (1) and (2), k represents Boltzmann’s constant, q represents the charge of an electron, and T represents temperature in Kelvin. On the semi-logarithmic I–V (ln(I)–V) graph, the intersection of the line at V = 0 represents the saturation current I₀. The Richardson constant is usually expressed by the symbol A; For p-type silicon, this value is approximately A*=32 A/cm^2^*K*^2^. V, *Φ*_*b*_ and A represent the applied voltage, effective potential barrier height (Schottky barrier height corresponding to the potential energy barrier at the metal–semiconductor interface), and surface area of ​​the diode, respectively.

The parameter n (ideality factor, which reflects the deviation from ideal thermionic emission behavior), was determined by Eq. (3) through the slope of the linear part of the ln(I)–V curve.

3 $$n = \frac{q}{{kT}}\frac{{dV}}{{d(\ln I)}}$$

Equation (4) given below can be used to calculate the Φ_b_ value.

4 $$\varphi _{b} = \frac{{kT}}{q}\ln \left( {\frac{{AA^{*} T^{2} }}{{I_{0} }}} \right)$$

**Fig. 5:**
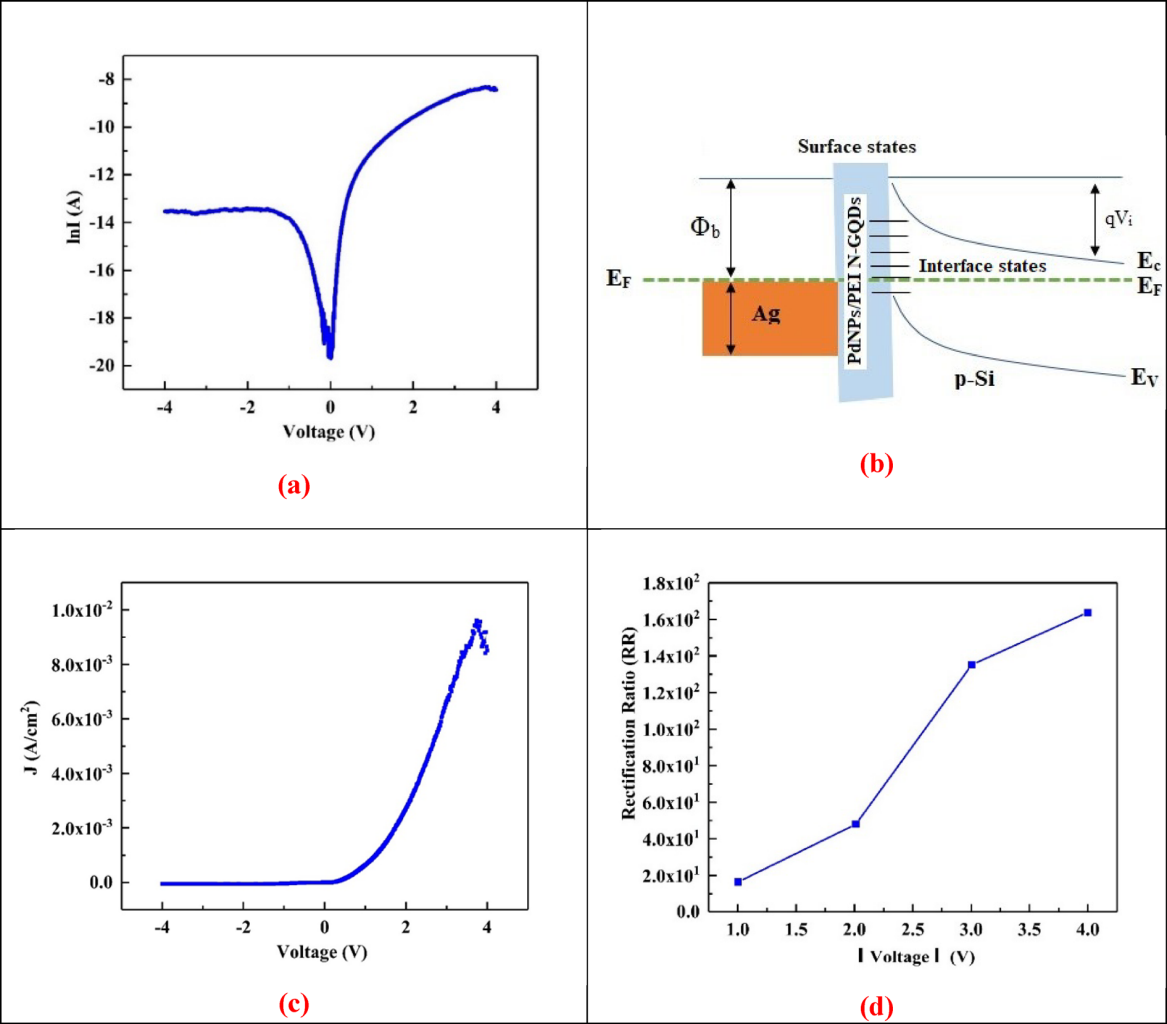
(**a**) ln(I)–V characteristics, (**b**) Equilibrium energy-band diagram, (**c**) Room-temperature J–V characteristic, and (**d**) Rectification Ratio at symmetric bias voltages (± 1 V, ± 2 V, ± 3 V, and ± 4 V) of the Ag/PdNPs/PEI N-GQDs/p-Si device.

The ln(I)–V characteristics of the Ag/PdNPs/PEI N-GQDs/p-Si structure, measured at 300 K under dark conditions within a ± 4 V range, are shown in Fig. 5a, since all diodes were primarily characterized at room temperature to provide a consistent baseline for comparison. The ln(I)–V characteristics of the Ag/PdNPs/PEI N-GQDs/p-Si structure, measured at 300 K under dark conditions within a ± 4 V range, are shown in Fig. 5a. ln(I)–V curve of the Ag/PdNPs/PEI N-GQDs/p-Si diode clearly demonstrates rectifying behavior. As seen in the plot, the forward current increases exponentially with voltage, while the reverse current remains nearly constant over the − 4 V to 0 V range. This asymmetry between forward and reverse bias indicates the successful formation of a Schottky junction. The sharp increase in current under forward bias and the suppressed current under reverse bias confirm the diode’s rectification capability. The low reverse leakage current also suggests good interface quality and minimal recombination at the junction. In the forward bias region, the ln(I)–V plot provides valuable insight into the electrical behavior of the Schottky diode. From the slope and intercept of the linear region, two key parameters can be extracted: n, which indicates how closely the diode follows ideal thermionic emission behavior, and the Φ_b_, representing the energy barrier at the metal–semiconductor interface. The n and Φ_b_ values ​​calculated according to the TE method were found to be 5.85 and 0.87 eV, respectively. A linear trend in this plot confirms that current transport is dominated by thermionic emission and validates the use of analytical methods to derive these parameters^36,38^.

To better understand the origin of the observed rectifying behavior, the energy band alignment of the Ag/PdNPs-doped PEI N-GQDs/p-Si heterostructure was analyzed. Figure 5b presents the equilibrium energy-band diagram of the structure. The rectifying characteristics primarily originate from the Ag/composite junction, where the difference between the work function of Ag (~ 4.5 eV) and the electronic structure of the PdNPs-doped PEI N-GQDs layer leads to the formation of a Schottky-type barrier. At the PdNPs-doped PEI N-GQDs nanocomposite/p-Si interface, the band gap of Si (1.12 eV) and that of the nanocomposite (~ 2.93 eV, estimated from the Tauc plot) establish a staggered (type-II) heterojunction that facilitates hole transport from Si into the nanocomposite. Surface states mainly originate from functional groups and Pd-induced defects on the GQDs, while interface states form at the nanocomposite/p-Si boundary due to dangling bonds and interfacial imperfections. These states can pin the Fermi level and alter the effective barrier height, thereby influencing the overall carrier transport.

The electrical performance of GQDs-based nanocomposite diodes was evaluated at 300 K to establish a consistent baseline for comparison across different doped GQDs-based material systems. Subsequent studies will incorporate variable-temperature measurements to provide further insights into barrier inhomogeneity and carrier transport mechanisms.

Figure 5c presents the room-temperature current density-voltage (J–V) characteristics of the Ag/PdNPs/PEI N-GQDs/p-Si. The current density remains near zero under reverse bias within the explored range, while it increases under forward bias. In the ~ 2–3.6 V interval, the semilog forward branch appears approximately linear, which is consistent with a TE–like regime. At higher biases (≈ 3.8–4 V), a slight deviation/roll-over is observed; this may arise from series resistance, local heating, or space-charge effects contributions.

Figure 5d shows the rectification ratio ($\:RR=\frac{{I}_{forward\:}}{\left|{I}_{reverse}\right|\:}\:$) evaluated at symmetric bias voltages. RR is lower at ± 1 V because forward transport is still limited by recombination/trap-assisted conduction, whereas reverse leakage remains relatively insensitive to bias; with larger |Voltage| (± 2 V, ± 3 V, and ± 4 V), forward current increases more steeply and RR rises.

The method developed by S.K. and N.W. Cheung was used to determine the diode parameters^39^. The R_s_ (series resistance, representing resistive losses in the diode) and n can be obtained from Eq. (5), while the Φ_b_ is determined from Eqs. (6) and (7).

5 $$\frac{{dV}}{{d(\ln I)}} = n\frac{{kT}}{q} + IR_{s}$$

6 $$H(I) = V - \frac{{nkT}}{q}\ln (\frac{I}{{AA^{*} T^{2} }})$$

7 $$H(I) = n\varphi _{b} + IR_{s}$$

**Fig. 6:**
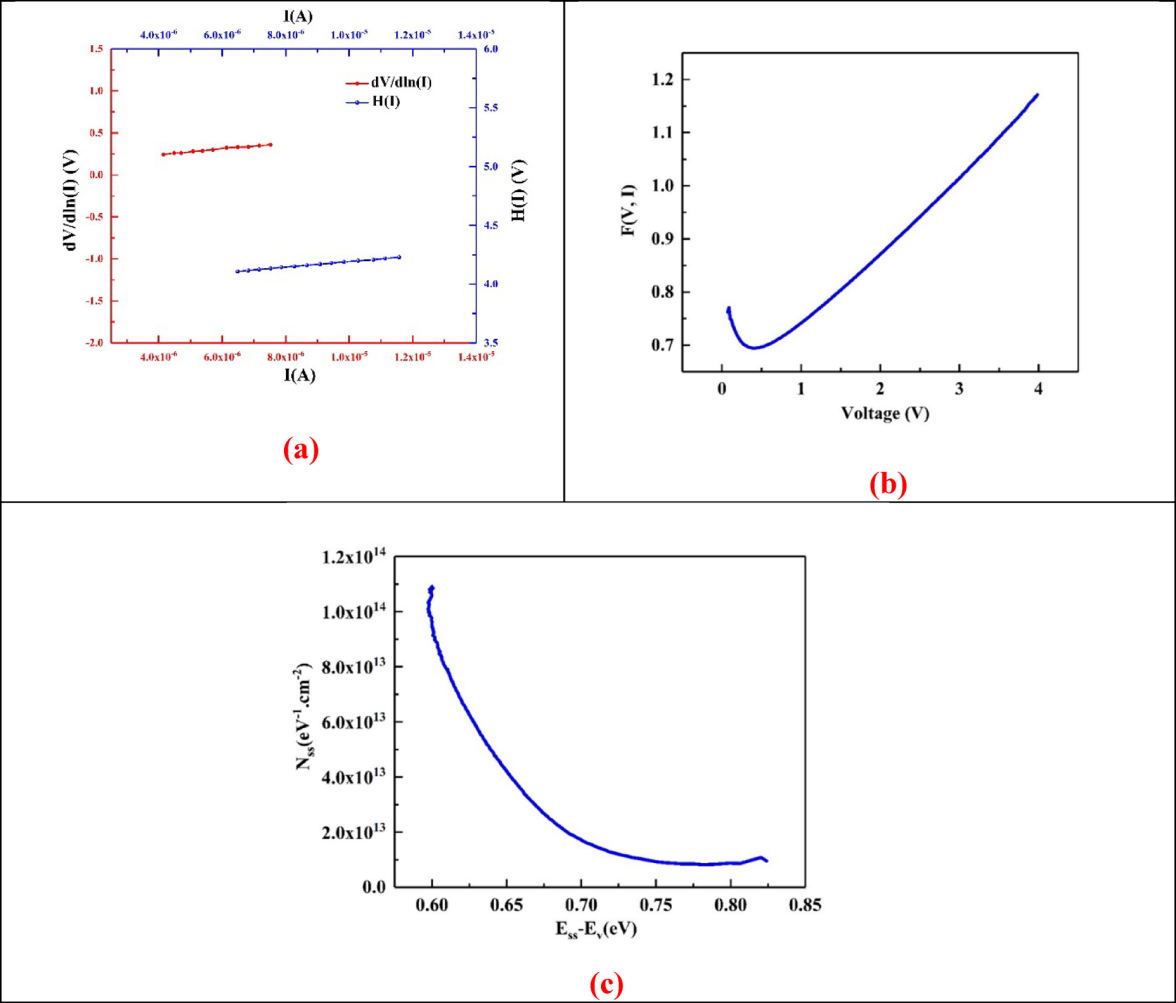
(**a**) dV/dln (I) vs. I and H(I) vs. I characteristics, (**b**) F(V, I) from Norde’s plot and (**c**) Energy-dependent profile of N_ss_ of the Ag/PdNPs/PEI N-GQDs/p-Si diode.

Figure 6a displays Cheung’s function plots of dV/dln(I) versus I and H(I) versus I for the Ag/PdNPs/PEI N-GQDs/p-Si diode in the dark at 300 K. The linearity observed in both graphs confirms the applicability of the TE model in the forward bias region. The slope of the dV/dln(I) plot yields n, while the H(I) plot provides information on the R_s_ and Φ_b_. The consistency and linear behavior of the data suggest good interface quality and a uniform distribution of potential barriers across the junction. The slopes of the curves obtained from Cheung’s analysis were used to determine the R_s_ of the diode. The calculated R_s_ values were 34.25 kΩ and 23.65 kΩ under dark conditions. The n was found to be 4.19 from the y-intercept of the dV/dln(I) versus I plot. Using this value of n, Φ_b_ was calculated as 0.68 eV. These values are in good agreement with the results obtained from the TE method.

In addition, the Norde function method has been used to determine R_s_ and Φ_b_^41^. The Norde function is first defined as Eq. (8), which relates the applied voltage to the current and device constants as follows:

8 $$\:F\left(V,I\right)=\frac{V}{\gamma\:}-\frac{kT}{q}\left[{ln}\left(\frac{I}{A{A}^{*}{T}^{2}}\right)\right]$$

The Φ_b_ is then calculated from Eq. (9), using the minimum of the F(V, I) curve. In the Norde analysis, the parameter γ was selected as 6, since it is the smallest integer greater than the extracted ideality factor (*n* ≈ 5.85), in accordance with the theoretical requirement that γ > n.

9 $$\:{\varnothing\:}_{b}=F\left({V}_{min}\right)+\frac{{V}_{min}}{\gamma\:}-\frac{kT}{q}$$

Similarly, the R_s_ is determined from Eq. (10), which depends on the slope parameter γ, n, and the corresponding current at the minimum point.

10 $$\:{R}_{s}=\frac{kT(\gamma\:-n)}{q{I}_{min}}$$

where I_min_ is the current that corresponds to the V_min_ value and F(V_min_) is the minimum point of the F(V, I)-V plot.

The F(V, I) versus voltage plot shown in Fig. 6b illustrates the Norde function, which is utilized to extract key diode parameters such as the Φ_b_ and R_s_. The minimum point of the curve corresponds to the voltage at which the barrier height is determined. The smooth, parabolic shape and distinct minimum indicate a reliable fit, supporting the precision and consistency of the parameters obtained through the Norde method^40^.

Considering the voltage-dependent n and Φ_b_, the energy-dependent change of surface trap density (N_ss_) using I–V data in the forward bias region was calculated with the help of Card–Rhoderick model^41^.

11 $$\:n\left(V\right)=qV/[kT{ln}\left(\frac{I}{{I}_{0}}\right)]$$

The voltage-dependent effective barrier height (Φ_e_) was evaluated using the following relation:

12 $$\:{\varPhi\:}_{e}=\varPhi\:\text{b}\text{o}+\left(1-\frac{1}{n\left(V\right)}\right)V$$

This expression accounts for the non-ideal behavior observed in real Schottky contacts, where interface states and barrier inhomogeneities cause deviations from the ideal thermionic emission model. Here, Φ_bo_ denotes the zero-bias barrier height, and n (V) represents the voltage-dependent ideality factor.

Furthermore, the energy position of the interface states for p-type semiconductors can be determined by the following expression^42^:

13 $$\:{E}_{ss}-{E}_{v}=q({\varPhi\:}_{e}-V)$$

This equation allows for the estimation of the energy position of surface states relative to the valence band edge, providing valuable insights into interface quality and trap behavior.

Thus, Eqs. (11), 12, and 13 were used to extract the N_ss_ vs. (E_ss_–E_v_) plot as follows^43,44^:

14 $$\:{N}_{ss}\left(V\right)=\frac{1}{q}\left[\frac{{\epsilon\:}_{i}}{d}\right(n\left(V\right)-1)-\frac{{\epsilon\:}_{s}}{{W}_{d}}]$$

This equation enables the evaluation of interface trap densities at various energy levels relative to the valence band. In Eq. (14), N_ss_(V) represents the voltage-dependent interface state density, which quantifies the density of localized energy states at the interfacial region between the nanocomposite layer and the semiconductor. The term ε_i_​ denotes the dielectric constant of the interfacial layer, while d corresponds to its physical thickness. ε_s_​ is the dielectric constant of the p-type silicon substrate, and w_d_​ indicates the depletion region width in the semiconductor. The ideality factor n(V), which varies with applied voltage, provides insight into the deviation from ideal diode behavior.

Figure 6c shows the variation of *N*_*ss*_ as a function of the energy difference (*E*_*ss*_*−E*_*v*_). The graph demonstrates a clear downward trend, indicating that the density of interface states decreases with increasing energy from the valence band edge. This behavior suggests a lower concentration of interface traps near the mid-gap, which is beneficial for improved charge transport and diode performance^45–47^. The smooth decay confirms the uniformity and quality of the interfacial layer formed by the PdNPs/PEI N-GQDs nanocomposites.

Table 1 summarizes the key diode parameters calculated at room temperature under dark conditions. The device exhibited an RR of ~ 1.64 × 10^2^ under ± 4 V, confirming its rectifying nature. The ideality factors obtained from the TE and Cheung methods ranged between 5.85 and 4.19, clearly deviating from ideal diode behavior. This deviation is most likely attributed to barrier inhomogeneity and interface states, as well as possible trap-assisted tunneling^48–50^. The Φ_b_ was method-dependent—0.87 eV (TE), 0.76 eV (Norde), and 0.68 eV (Cheung)—highlighting the sensitivity of parameter determination to the analysis technique. Similarly, the R_s_ varied depending on the method used, with values of 110.17 kΩ (Norde), 34.25 kΩ (Cheung’s dV/dlnI vs. I), and 23.65 kΩ (Cheung’s H(I) vs. I). Taken together, these results indicate Schottky behavior dominated by barrier height fluctuations and interface-related states, rather than by a uniform barrier distribution.

**Table 1:** Key diode parameters of the Ag/PdNPs/PEI N-GQDs /p-Si diode.

		Ag/PdNPs/PEI *N*-GQDs /*p*-Si		
RR (± 4 V)		1.64 × 10^2^		
Methods		n	Φ_b_ (eV)	R_s_ (kΩ)
TE		5.85	0.87	–
Norde		–	0.76	110.17
Cheung	dV/dlnI vs. I	4.19	–	34.25
	H(I) vs. I	-	0.68	23.65

Table 2 summarizes the comparative electrical performance of the Pd/PEI N-GQDs-based diode and selected Schottky diodes from previous studies. According to the comparative data presented in Table 2, the PdNPs/PEI N-GQDs-based Schottky diode demonstrates a balanced performance relative to other GQD-based diode structures reported in the literature. Although the ideality factor of the present device (*n* = 5.85) is relatively high, which may indicate the presence of interface traps or barrier inhomogeneities, the diode still exhibits a favorable barrier height (Φb = 0.87 eV, TE method), supporting efficient rectification. Compared to the Al/PEI N-GQDs/p-Si diode reported in^18^, which exhibited a much higher rectification ratio (~ 2.8 × 10^4^), the PdNPs/PEI N-GQDs-based diode demonstrated more moderate performance (RR = 1.64 × 10^2^), although it remains comparable to or even superior to other reported devices, such as the Ag/4A-MORP N-GQDs/p-Si structure^13^, possibly due to differences in interface engineering and doping efficiency. Furthermore, R_s_ values obtained by different methods remain within a reasonable range, indicating stable carrier transport characteristics. Compared to lanthanide-doped counterparts, such as La(OH)_2_^20^—or Gd-doped GQDs^35^, the Pd-based diode achieves a more moderate yet consistent electrical profile. These results suggest that PdNPs/PEI N-GQDs nanocomposites can serve as a promising interfacial layer for Schottky diode applications, particularly in rectification-focused and potentially photoresponsive devices.

**Table 2:** Comparison of diode parameters for various types of Schottky diodes.

Diodes	*n*	Φ_b_ (eV)	*R*_s_(kΩ)	RR	References
Ag/PdNPs/ PEI N-GQDs /p-Si	5.85 (TE) 4.19 (Cheung)	0.87 (TE) 0.76 (Norde) 0.68 (Cheung)	110 (Norde) 34.25 (Cheung) 23.65 (Cheung)	1.64 × 10^2^ at ± 4 V	Present study
Ag/4A-MORP N-GQDs /p Si	6.7 (TE) 3.07 (Cheung)	0.74 (TE) 0.80 (Norde) 0.71 (Cheung)	50 (Norde) 330 (Cheung) 166 (Cheung)	1.02 × 10^2^ at ± 3 V	^13^
Al/ La(OH)_3_NPs-doped PEI N-GQDs/p-Si (110 mW/cm^2^)	2.43 (TE) 3.46 (Cheung)	0.76 (TE) 0.69 (Cheung)	4.8 (TE) 6.9-4.0 (Cheung)	1.52 × 10^2^ at ± 2 V	^20^
Al/ La(OH)_3_NPs-doped PEI N-GQDs/p-Si (Dark)	2.8 (TE) 3.31 (Cheung)	0.74 (TE) 0.70 (Cheung)	4.8 (TE) 6.9–4.3 (Cheung)	2.8 × 10^3^ at ± 2 V	^20^
Al/PEI N-GQDs /p Si	3.71 (TE) 9.20 (Cheung)	0.76 (TE) 0.82 (Norde) 0.66 (Cheung)	5450 (Norde) 143 (Cheung) 110 (Cheung)	2.8 × 10^4^ at ± 5 V	^18^
Al/GdNPs/PEI N-GQDs/p-Si	4.9 (TE) 11.6 (Cheung)	0.605 (TE) 0.595 (Norde) 0.584 (Cheung)	3.85 (TE) 2.63–2.35 (Cheung) 3.35 (Norde)	14 at ± 5 V	^36^

## Discussion

The results of this study demonstrate that Pd doping in PEI-functionalized N-GQDs effectively tunes their optical and electronic properties for diode applications. The PdNPs/PEI N-GQDs interlayer enabled the Ag/p-Si heterostructure to exhibit a rectification ratio of approximately 1.64 × 10^2^ at ± 4 V and Schottky-type transport characterized by barrier heights of 0.87, 0.76, and 0.68 eV, depending on the analysis method. The broad PL emission around 580 nm indicates defect-related recombination consistent with the modified band structure induced by Pd incorporation. The proposed band alignment shows that the rectifying behavior originates from the Ag/composite Schottky barrier, while a type-II staggered heterojunction at the composite/p-Si interface facilitates hole transport. These findings clarify the charge transport mechanism in doped GQD-based heterostructures and highlight their potential for future carbon-based nanoelectronic and optoelectronic devices.

## Methods

### Materials

All reagents used in this study—including citric acid (CA), branched polyethyleneimine (PEI; average Mn ~ 10,000 by GPC, average Mw ~ 25,000 by LS), and palladium nitrate dihydrate [Pd(NO_2_)_2_·2 H_2_O]—were obtained from Sigma-Aldrich and utilized as received, without further purification.

### Synthesis of PEI N-GQDs and PdNPs/PEI N-GQDs nanocomposites

The PEI N-GQDs and PdNPs/PEI N-GQDs nanocomposites were synthesized through a hydrothermal approach, as previously reported^18,33–35^. Initially, 5.78 g of CA monohydrate and 6.55 g of branched polyethyleneimine (PEI) were completely dissolved in 50 mL of deionized water. This solution was transferred into a Teflon-lined autoclave and subjected to thermal treatment in an oven at 200 °C for 18 h. After naturally cooling to ambient temperature, the resulting suspension was centrifuged at 12,000 rpm for 10 min to isolate the nanoparticles. The collected PEI N-GQDs were subsequently rinsed twice with deionized water and once with ethanol, followed by drying in a vacuum oven. The final product was stored in a desiccator to prevent moisture absorption.

For the synthesis of PdNPs/PEI N-GQDs nanocomposites, 0.10 g of the as-prepared PEI N-GQDs was added to 50 mL of ultrapure water in a 250 mL round-bottom flask. A separate solution containing 0.10 g of Pd(NO_2_)_2_·H_2_O in 50 mL of water was then introduced into the flask. The combined mixture was stirred and heated in a water bath at 90 °C for 2 h. A gradual color shift from yellow to gray-black was observed, indicating the complete reduction of Pd^2+^ ions to elemental Pd⁰ nanoparticles. The mixture was then filtered, and the obtained solid was washed thoroughly with deionized water (twice) to remove residual byproducts. The purified product was converted to powder form and vacuum-dried. The resulting PdNPs/PEI N-GQDs nanocomposites were stored in a desiccator for later applications (Fig. 1a).

### Device architecture

Prime CZ-Si wafers (3 inch, thickness 380 ± 20 μm, (100), 1-side polished, p-type (Boron), TTV < 10 μm, 1–10 Ω·cm) served as substrates. Acceptor density was estimated as NA ≈ (1–3) × 10^16^ cm^−2^ at ρ ≈ 1 Ω cm and NA ≈ (1–3) × 10^15^ cm^-2^ at ρ ≈ 10 Ω cm. A 124 nm Ag film was thermally evaporated onto the rear side of p-Si, followed by rapid thermal annealing at 450 °C for 5 min under high vacuum (~ 1 × 10^−5^ mbar) to enhance ohmic contact. Prior to spin-coating the PdNPs/PEI N-GQDs layer, native oxide was removed from the substrate using a 2% hydrofluoric acid (HF) solution. The backside and edges were protected with photoresist and Kapton tape during HF cleaning. After cleaning, protective layers were removed, and the front surface was left hydrogen-terminated. The PdNPs/PEI N-GQDs nanocomposite was then spin-coated at 3000 rpm for 30 s, forming a uniform ~ 30 nm film with minimal air exposure. This thickness was maintained throughout the study for consistency and reproducibility. Circular top contacts were defined by thermally depositing an additional 124 nm Ag layer through a 1.8 mm aperture shadow mask. The device architecture is shown in Fig. 1b.

### Characterization techniques

The structural and chemical properties of PdNPs/PEI N-GQDs nanocomposites were examined using Fourier Transform Infrared (FT-IR) Spectroscopy, Ultraviolet-Visible (UV–Vis) Spectroscopy, X-ray Photoelectron Spectroscopy (XPS), Transmission Electron Microscopy (TEM), and Photoluminescence (PL) Spectroscopy.

FTIR spectra were collected in the range of wavenumbers (cm^−1^) using a Perkin Elmer BX II spectrometer, employing KBr pellets as the sample medium. UV–Vis absorption spectra were acquired with a PG Instruments T + 80 spectrophotometer. The morphological features of the PdNPs/PEI N-GQDs nanocomposites were examined via TEM using a FEI Tecnai G2 Twin microscope operating at 200 kV. To minimize nanoparticle agglomeration, aqueous suspensions of the nanocomposites were ultrasonicated prior to sample preparation. A 10 µL aliquot of the suspension was deposited onto 200-mesh copper grids coated with formvar and carbon films, followed by air-drying. Elemental composition analysis of the PdNPs/PEI N-GQDs nanocomposites was carried out using XPS, employing a Bruker AXs XFlash 4010 detector. PL was conducted by the excitation of a Kimmon IK series He-Cd continuous wave laser with a 325 nm wavelength.

The current–voltage (I–V) characteristics of the Ag/PdNPs/PEI N-GQDs/p-Si Schottky diode were measured using a Keithley 4200-SCS semiconductor characterization system integrated with a Keithley 2400 source meter. The measurements were carried out in the voltage range of − 4 V to + 4 V, with a step size of 0.01 V and a dwell time of approximately 100 ms at each point. A compliance current of 1 mA was applied to protect the diode. All measurements were conducted at room temperature (300 K) under dark conditions.

## Data Availability

All data included in this study are available upon request by contact with the corresponding author (E.D).
